# Supplementary material for: A low-threshold potassium current enhances sparseness and reliability in a model of avian auditory cortex
Source: PLoS Comput Biol. 2019 Jan 28;15(1):e1006723. doi: 10.1371/journal.pcbi.1006723 (PMC6366721; doi:10.1371/journal.pcbi.1006723)
Supplement: S1 Appendix — (PDF) [file pcbi.1006723.s001.pdf]

S1 Appendix: Model equations.

The somatic CM model consists of a single, isopotential compartment with capacitance C_m. The dynamics of the voltage are described by current conservation,

$$C_m \frac{dV(t)}{dt} = I_{Na} + I_A + I_{HT} + I_{LT} + I_h + I_{leak} + I_{stim}(t) + I_{noise}(t). \quad (1)$$

Each of the currents is modeled as an ohmic conductance with zero or more gating variables. The dynamics of the gating variables obey first-order kinetics:

$$\frac{dx(t)}{dt} = \frac{x^{\infty}(V(t)) - x(t)}{\tau_x(V(t))}, \quad (2)$$

where $x^{\infty}(V)$ gives the equilibrium activation and $\tau_x(V)$ gives the time constant as functions of voltage. The equations for the currents and kinetic functions generally follow Rothman & Manis [1], but with some adjustments to half-activation voltages and time constants to match the spike threshold and shape in CM.

Transient Sodium

$$\begin{aligned}
I_{Na} &= \bar{g}_{Na} m_{Na}^3 h_{Na} (E_{Na} - V_m) \\
m^{\infty}(V) &= [1 + \exp((-41 - V)/7)]^{-1} \\
\tau_m(V) &= 0.077 + [0.26 \exp((V + 63)/18) + 1.87 \exp(-(V + 63)/25)]^{-1} \\
h^{\infty}(V) &= [1 + \exp(-(-68 - V)/6)]^{-1} \\
\tau_h(V) &= 1.15 + [0.036 \exp((V + 63)/11) + 0.051 \exp(-(V + 63)/25)]^{-1}
\end{aligned} \quad (3)$$

High-threshold Potassium

$$\begin{aligned} I_{HT} &= \bar{g}_{HT}(0.85m_{HT}^2 + 0.15n_{HT})(E_K - V_m), \\ m^{\infty}(V) &= [1 + \exp((-11 - V)/5)]^{-1} \\ \tau_m(V) &= 1.35 + [0.057\exp((V + 60)/24) + 0.11\exp(-(V + 60)/23)]^{-1} \\ n^{\infty}(V) &= [1 + \exp((-19 - V)/6)]^{-1} \\ \tau_h(V) &= 9.65 + [0.021\exp((V + 60)/32) + 0.026\exp(-(V + 60)/22)]^{-1} \end{aligned} \tag{4}$$

A-type Potassium

$$\begin{aligned} I_A &= \bar{g}_A m_A^4 h_A c_A(E_K - V_m), \\ m^{\infty}(V) &= [1 + \exp((-31 - V)/7)]^{-1/4} \\ \tau_m(V) &= 0.193 + [0.036\exp((V + 60)/14) + 0.15\exp(-(V + 60)/24)]^{-1} \\ h^{\infty}(V) &= [1 + \exp(-(-66 - V)/7)]^{-1/2} \\ \tau_h(V) &= 1.93 + [0.0073\exp((V + 60)/27) + 0.051\exp(-(V + 60)/24)]^{-1} \\ c^{\infty}(V) &= h^{\infty}(V) \\ \tau_c(V) &= 19.3 + 174 * [1 + \exp(-(V + 66)/17)]^{-1} \end{aligned} \tag{5}$$

Low-threshold Potassium

$$\begin{aligned} I_{LT} &= \bar{g}_{LT} m_{LT}^4 h_{LT}(E_K - V_m), \\ m^{\infty}(V) &= [1 + \exp((-48 - V)/6)]^{-1/2} \\ \tau_m(V) &= 2.9 + [0.031\exp((V + 60)/6) + 0.083\exp(-(V + 60)/45)]^{-1} \\ h^{\infty}(V) &= (1 - \zeta)[1 + \exp(-(-71 - V)/10)]^{-1} + \zeta \ (\zeta = 0.5) \\ \tau_h(V) &= 96.5 + 1000[0.52\exp((V + 60)/20) + 0.52\exp(-(V + 60)/8)]^{-1} \end{aligned} \tag{6}$$

Hyperpolarization-activated cation current

$$\begin{aligned} I_h &= \bar{g}_h h_h(E_h - V_m), \\ h^{\infty}(V) &= [1 + \exp(-(-76 - V)/7)]^{-1/2} \\ \tau_h(V) &= 48.25 + 10^5[123\exp((V + 60)/12) + 8.8\exp(-(V + 60)/14)]^{-1} \end{aligned} \tag{7}$$

Leak current and consensus parameters

The leak current is passive: $I_{leak} = g_{leak}(E_{leak} - V)$. In the consensus model, $\bar{g}_{Na} = 750$ nS, $E_{Na} = 40$ mV, $\bar{g}_{HT} = 95$ nS, $\bar{g}_A = 30$ nS, $E_K = -82$ mV, $\bar{g}_h = 0.5$ nS, $E_h = -43$ mV, $\bar{g}_{leak} = 1.3$ nS, and $E_{leak} = -75$ mV.

References

1. Rothman JS, Manis PB. The roles potassium currents play in regulating the electrical activity of ventral cochlear nucleus neurons. Journal of Neurophysiology. 2003;89(6):3097–3113.
